# Comparative Experimental Raman, DFT, and Chemometric Characterization of Selected Phenolic Acids: Structural and Environmental Contributions to the Vibrational Response

**DOI:** 10.3390/molecules31152667

**Published:** 2026-07-31

**Authors:** Jose Alfonso Prieto Palomo, Juan Lopez-Martinez, Joaquín Alejandro Hernández Fernández

**Affiliations:** 1 Chemistry Program, Department of Natural and Exact Sciences, San Pablo Campus, Universidad de Cartagena, Cartagena de Indias D.T. y C., Cartagena 130015, Colombia; 2 Institute of Materials Technology (ITM), Universitat Politecnica de Valencia (UPV), Plaza Ferrandiz and Carbonell s/n, 03801 Alcoy, Spain; jlopezm@mcm.upv.es; 3 Department of Natural and Exact Science, Universidad de la Costa, Barranquilla 080002, Colombia

**Keywords:** phenolic acids, Raman spectroscopy, density functional theory, vibrational assignment, molecular electrostatic potential, HOMO–LUMO analysis, principal component analysis

## Abstract

Phenolic acids exhibit structure-dependent vibrational responses governed by aromatic substitution, π-conjugation, oxygenated functional groups, and molecular environment. In this work, p-coumaric, caffeic, trans-ferulic, and gallic acids were investigated through an integrated experimental Raman, density functional theory (DFT), and chemometric approach to establish molecular relationships between hydroxylation, methoxylation, conjugation, and Raman spectral behavior. Raman spectra were recorded in the solid state and in an ethanol/water (1:1, *v*/*v*) mixture. At the same time, DFT calculations were used to optimize the molecular structures, simulate Raman spectra, assign vibrational modes, and evaluate molecular electrostatic potential, frontier orbitals, electronic descriptors, and localized orbital locator maps. The solid-state Raman spectra provided the most resolved molecular fingerprints, with hydroxycinnamic acids exhibiting intense bands corresponding to aromatic and conjugated ν(C=C) modes. In contrast, gallic acid displayed a distinct hydroxybenzoic vibrational pattern dominated by phenolic C–O/O–H and carboxylic contributions. DFT-assisted assignments confirmed that the main spectral differences arise from coupled vibrations involving ν(C=C), ν(C=O), ν(C–O), δ(O–H), aromatic ring deformations, and methoxy-related modes. Molecular electrostatic potential (MEP) and localized orbital locator (LOL) analyses showed that oxygen-centered regions are the most electrostatically and electronically localized sites, thereby explaining the sensitivity of C–O, O–H, and C=O bands to solvent-mediated interactions. HOMO–LUMO analysis revealed extended frontier-orbital delocalization in hydroxycinnamic acids, in contrast to the more localized hydroxybenzoic electronic structure of gallic acid. Principal component analysis confirmed that solid-state Raman spectra provide stronger chemometric discrimination than solution spectra, with PC1 and PC2 explaining 79.0% of the total variance in the solid-state dataset. Overall, the results show that specific functional groups define the principal vibrational domains of the studied phenolic acids. In contrast, the exact band positions, relative intensities, and coupling patterns are additionally modulated by aromatic substitution, π-conjugation, electronic distribution, physical state, and molecular environment. Within the limitations of a single-conformer isolated-molecule model, the combined Raman–DFT–chemometric approach provides a comparative interpretation of the vibrational fingerprints of the four selected compounds.

## 1. Introduction

Phenolic acids constitute a broad family of aromatic compounds widely distributed in plant matrices, foods, natural extracts, and antioxidant systems [1,2]. Their structures contain hydroxyl, methoxy, and carboxyl groups whose number and position influence electronic distribution, hydrogen-bonding capacity, antioxidant behavior, and vibrational response [3,4]. In this study, p-coumaric, caffeic, trans-ferulic, and gallic acids were selected as representative naturally occurring phenolic acids with distinct substitution patterns and molecular frameworks [5,6]. p-Coumaric acid contains one phenolic hydroxyl group and the conjugated Ar–CH=CH–COOH backbone [7]; caffeic acid retains this framework but incorporates a second hydroxyl group, forming a catechol moiety [8]; and trans-ferulic acid contains both hydroxyl and methoxy substituents, introducing additional C–O and –OCH_3_-related vibrational contributions [9]. Gallic acid, in contrast, is a trihydroxylated hydroxybenzoic acid that lacks the conjugated vinyl side chain characteristic of hydroxycinnamic acids [10].

The molecular set was intentionally selected to include three hydroxycinnamic acids sharing the same conjugated backbone, along with gallic acid as an external hydroxybenzoic acid contrast. Accordingly, p-coumaric, caffeic, and trans-ferulic acids provide the most internally consistent comparison of hydroxyl- and methoxy-related spectral variations. Gallic acid was not included as a direct analog for isolating π-conjugation, but rather to examine how a highly hydroxylated and structurally different molecular backbone modifies the Raman fingerprint. Comparisons involving gallic acid, therefore, reflect simultaneous changes in substitution patterns, conjugation, and molecular topology.

Raman spectroscopy is particularly suitable for characterizing these compounds because it is sensitive to changes in molecular polarizability [11,12]. Bands involving ν(C=O), ν(C=C), ν(C–O), δ(O–H), aromatic-ring deformations, and C–H vibrations provide information about the main structural fragments of phenolic acids [13,14]. Nevertheless, these signals cannot always be treated as fully isolated functional-group vibrations because several normal modes involve coupled displacements of the aromatic ring, carboxylic group, hydroxyl groups, and methoxy substituents [15]. Thus, specific functional groups define the principal vibrational regions, whereas their exact positions, relative intensities, and coupling patterns are further influenced by the overall molecular framework and the surrounding environment.

Density functional theory provides a complementary approach for examining normal modes, supporting Raman band assignments, and relating spectral differences to molecular geometry and electronic descriptors [16,17]. Comparison between experimental Raman shifts and scaled harmonic frequencies helps distinguish vibrations dominated by aromatic or conjugated coordinates from those involving carboxylic, hydroxyl, or methoxy groups [18,19]. However, isolated-molecule calculations do not explicitly reproduce crystal packing, intermolecular hydrogen bonding, solvent interactions, or the full conformational ensemble. Previous vibrational, crystallographic, theoretical, and high-resolution rotational studies have shown that phenolic acids may exhibit multiple stable conformers. Eight gas-phase structures have been identified for caffeic acid, whereas two planar rotamers of gallic acid, differing mainly in the orientation of the carboxylic group, have been experimentally detected. Similarly, rotational spectroscopy established a conformational map comprising two conformers of trans-cinnamic acid and four conformers of trans-p-coumaric acid [20,21,22].

Accordingly, this work presents a comparative experimental study of Raman, DFT, and chemometrics on the four selected phenolic acids. Principal component analysis was applied as an exploratory tool to examine spectral similarities, differences, and replicate dispersion rather than as a validated classification model [23,24]. The contribution of the study lies in integrating solid-state and ethanol/water Raman spectra, DFT-assisted assignments, electronic descriptors, and exploratory PCA within a common framework. The selected compounds do not constitute a factorial series in which hydroxylation, methoxylation, and conjugation are independently varied. Cinnamic acid would provide a suitable unsubstituted reference for isolating aromatic-substitution effects while preserving the Ar–CH=CH–COOH backbone; however, it was not included in the original experimental design. Consequently, the conclusions are restricted to comparative structure–vibrational trends within the investigated molecular set rather than to the independent quantification of individual substituent or conjugation effects.

## 2. Results and Discussion

### 2.1. Solid-State Raman Spectra of Phenolic Acids

**Figure 1:**
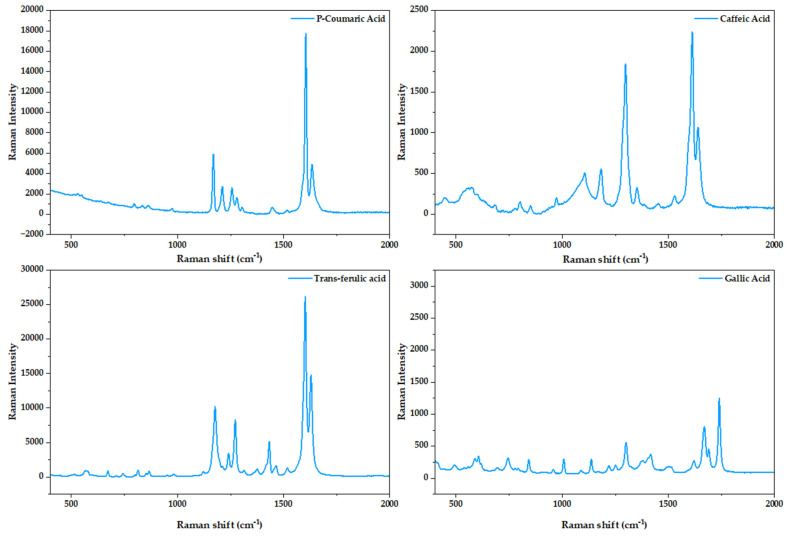
Solid-state Raman spectra of p-coumaric, caffeic, trans-ferulic, and gallic acids recorded in the 400–2000 cm^−1^ region. Each spectrum is presented using its corresponding intensity scale; therefore, absolute Raman intensities should not be directly compared among compounds.

The solid-state Raman spectra of p-coumaric, caffeic, trans-ferulic, and gallic acids are shown in Figure 1. The spectral region between 400 and 1800 cm^−1^ was selected as the main vibrational window because it contains the most structurally informative bands associated with aromatic ring deformation, ν(C=C) stretching, ν(C=O) stretching, ν(C–O) vibrations, δ(O–H) modes, and coupled vibrations involving the phenolic and carboxylic groups. To ensure the reproducibility and repeatability of the measurements, each sample was analyzed five times under identical experimental conditions, and the spectra presented here correspond to the average of the five replicates. In general, the spectra exhibited well-defined Raman bands, confirming that the solid-state measurements provide clear molecular fingerprints for the studied phenolic acids.

The three hydroxycinnamic acids, p-coumaric, caffeic, and trans-ferulic acids, displayed characteristic Raman bands in the high-wavenumber region of the fingerprint window, particularly between approximately 1550 and 1650 cm^−1^. These bands are mainly associated with aromatic ν(C=C) stretching and with vibrations coupled to the conjugated C=C–COOH side chain. This behavior is consistent with the presence of an extended π-conjugated system connecting the aromatic ring and the carboxylic group. The spectral profile of trans-ferulic acid contained prominent and well-resolved bands in the 1150–1300 cm^−1^ and 1550–1650 cm^−1^ regions. The former region is expected to include contributions from ν(C–O), δ(O–H), aromatic C–H in-plane deformation, and modes related to the methoxy substituent. In contrast, the latter is dominated by aromatic and conjugated C=C stretching vibrations.

The spectrum of p-coumaric acid was characterized by a relatively simple profile, with a dominant band in the region close to 1600 cm^−1^ and additional bands in the 1150–1300 cm^−1^ range. This profile is consistent with a hydroxycinnamic structure containing one phenolic hydroxyl group and a conjugated side chain. In contrast, caffeic acid exhibited a more diffuse spectral pattern, with intense contributions in the 1250–1300 cm^−1^ and 1550–1650 cm^−1^ regions. The increased complexity of the caffeic acid spectrum can be attributed to the presence of two adjacent phenolic hydroxyl groups, which introduce additional C–O/O–H vibrational contributions and favor stronger coupling between phenolic and aromatic ring modes.

Trans-ferulic acid exhibited a well-defined spectral profile characterized by bands associated with aromatic ring vibrations, conjugated ν(C=C) stretching, and C–O-related modes. The methoxy group introduced additional vibrational contributions in the C–O stretching and C–H deformation regions, thereby differentiating its fingerprint from those of p-coumaric and caffeic acids. Consequently, the spectral distribution of trans-ferulic acid reflects contributions from both the hydroxycinnamic backbone and methoxy-coupled vibrational coordinates.

Gallic acid exhibited a markedly different Raman profile from the hydroxycinnamic acids. Several bands were distributed across the 400–1800 cm^−1^ region, producing a more dispersed vibrational pattern than that observed for the hydroxycinnamic compounds. This difference is chemically relevant because gallic acid is a hydroxybenzoic acid and does not contain the conjugated vinylene C=C–COOH chain present in the hydroxycinnamic acids. Consequently, its Raman fingerprint is mainly associated with the substituted aromatic ring, the carboxylic group directly attached to the ring, and the three phenolic hydroxyl groups. The distinct band distribution therefore supports the influence of the molecular backbone and substitution pattern on the vibrational response.

The solid-state Raman spectra revealed clear differences among the phenolic acids studied. The hydroxycinnamic acids exhibited characteristic bands associated with the conjugated aromatic system, whereas gallic acid showed a distinct vibrational fingerprint associated with the hydroxybenzoic framework. These results indicate that solid-state Raman spectroscopy provides an appropriate experimental basis for comparing the structural characteristics of the selected phenolic acids. In addition, because the solid-state spectra are less affected by solvent contributions, they represent the primary experimental reference for comparison with the DFT-calculated Raman spectra and for subsequent vibrational assignments.

### 2.2. Raman Spectra in Solution and Solvent Effects

**Figure 2:**
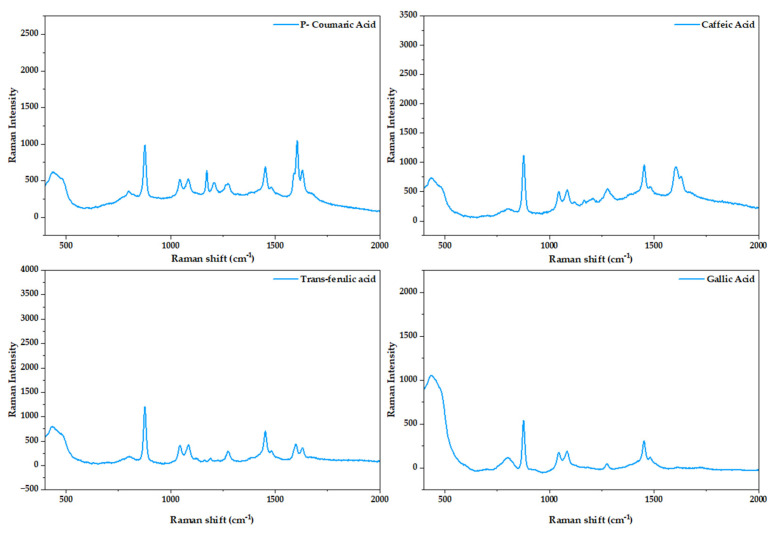
Raman spectra of p-coumaric, caffeic, trans-ferulic, and gallic acids in ethanol: water solution recorded in the 400–2000 cm^−1^ region.

The Raman spectra of p-coumaric, caffeic, trans-ferulic, and gallic acids in ethanol: water solution are shown in Figure 2. Compared with the solid-state spectra, the solution spectra exhibited a noticeable decrease in Raman intensity and a broader spectral background. This behavior is expected because the analytes are molecularly dispersed in the solvent, which reduces the number of scattering molecules within the laser focal volume and introduces additional spectral contributions from the ethanol: water medium. Consequently, the solution spectra should be interpreted as vibrational responses affected by solvation, dilution, and solvent–solute interactions rather than as direct equivalents of the solid-state spectra.

In all phenolic acid solutions, the most relevant spectral information remained within the 400–1800 cm^−1^ region. However, several bands that were clearly resolved in the solid state became weaker or partially overlapped in solution. This effect was particularly evident for low-intensity bands associated with phenolic ν(C–O), δ(O–H), aromatic C–H deformation, and coupled ring modes. The broad contribution observed in the low-wavenumber region and the presence of intense features around the fingerprint region indicate that the ethanol: water solvent system contributes to the recorded spectra. Therefore, the solvent blank must be considered during interpretation, especially in regions where solvent bands may overlap with weak analyte vibrations.

The spectra of the hydroxycinnamic acids, p-coumaric, caffeic, and trans-ferulic acids, retained characteristic bands related to the aromatic ring and the conjugated C=C–COOH side chain. Nevertheless, their intensities were markedly lower than in the solid state, and some bands appeared broadened. This broadening can be attributed to the distribution of solute conformations and microenvironments in solution, as well as to hydrogen-bonding interactions with ethanol and water molecules. These interactions are particularly relevant for the carboxylic acid and phenolic hydroxyl groups, which can act as hydrogen-bond donors and acceptors.

For p-coumaric acid, the solution spectrum maintained distinguishable bands in the aromatic and conjugated regions, although with lower intensity and reduced spectral definition compared with the solid-state spectrum. The bands associated with ν(C=C) aromatic/conjugated vibrations and ν(C–O)/δ(O–H) contributions were still observable, indicating that the hydroxycinnamic backbone remains spectroscopically identifiable in solution. However, the lower intensity and partial band broadening suggest that solvent interactions weaken the direct correspondence between the solution spectrum and the solid-state vibrational fingerprint.

Caffeic acid also exhibited a solution spectrum with defined but broadened features. The presence of two adjacent phenolic hydroxyl groups increases the likelihood of hydrogen bonding with the ethanol–water medium, which can affect the bands associated with ν(C–O), δ(O–H), and coupled aromatic modes. As a result, the solution spectrum of caffeic acid showed a more distributed profile than that expected for a single dominant vibrational contribution. This behavior is consistent with the higher sensitivity of catechol-type structures to solvent-mediated interactions.

The solution spectrum of trans-ferulic acid showed broader and less clearly resolved features than its solid-state spectrum. Nevertheless, bands associated with aromatic/conjugated vibrations and C–O-related modes remained detectable. The methoxy substituent contributes to specific vibrations in the fingerprint region, although in solution these contributions may partially overlap with solvent bands or other ring-associated modes. Therefore, the methoxy-related features remain identifiable but are less sharply resolved than in the solid-state spectrum.

Gallic acid presented a distinct solution behavior among the studied compounds. Its spectrum showed a pronounced background contribution and analyte bands with limited spectral definition. This response is consistent with its hydroxybenzoic structure, which bears three phenolic hydroxyl groups that can interact with the ethanol/water medium through hydrogen bonding. Such interactions may broaden or shift bands related to ν(C–O), δ(O–H), and the carboxylic group. In addition, the absence of the vinylene C=C–COOH chain present in hydroxycinnamic acids produces a different distribution of conjugated Raman-active modes, contributing to the reduced definition of some spectral features.

The differences between the solid-state and solution spectra indicate that solvation significantly affects the Raman response of phenolic acids. The bands associated with the aromatic framework are generally more stable. In contrast, those involving the carboxylic group, phenolic C–O bonds, and O–H-related vibrations are more sensitive to the solvent environment. This sensitivity is chemically reasonable because ethanol and water can form hydrogen-bonding interactions with –COOH and –OH groups, leading to band shifts, broadening, and changes in intensity. Therefore, a perfect overlap between solid-state and solution Raman spectra should not be expected.

The Raman spectra in solution complement the solid-state analysis by showing how the molecular environment modulates the vibrational response of phenolic acids. While the solid-state spectra provide the clearest fingerprints for DFT comparison and vibrational assignment, the solution spectra reveal the influence of solvation and hydrogen bonding on the experimental Raman response. This distinction is essential for correctly interpreting the spectroscopic behavior of phenolic acids under different physical states.

### 2.3. DFT-Optimized Geometries and Structural Features

**Figure 3:**
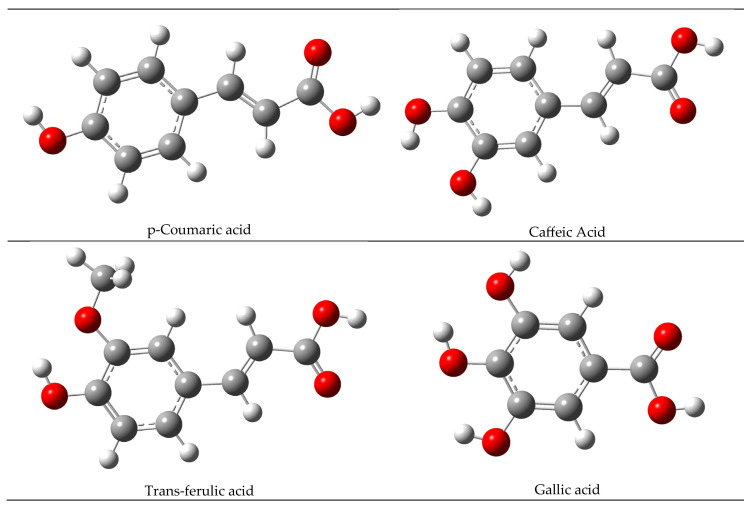
DFT-optimized geometries of p-coumaric, caffeic, trans-ferulic, and gallic acids.

The geometries discussed in this section correspond to one optimized conformer selected for each neutral molecule. No exhaustive conformational search was performed. Therefore, the reported structural parameters should be interpreted as descriptors of the selected computational models, not as a complete description of the compounds’ conformational landscapes. The DFT-optimized geometries of p-coumaric, caffeic, trans-ferulic, and gallic acids are shown in Figure 3. All optimized structures corresponded to real minima on the potential energy surface, as confirmed by the absence of imaginary vibrational frequencies. The optimized geometries provide a structural basis for interpreting the experimental Raman spectra, particularly the bands associated with the carboxylic group, phenolic C–O bonds, methoxy substitution, and the conjugated C=C–COOH side chain.

The three hydroxycinnamic acids, p-coumaric, caffeic, and trans-ferulic acids, retained a highly planar conjugated backbone after geometry optimization. The ring–side-chain dihedral angles were close to ±180°, indicating an almost coplanar arrangement between the aromatic ring and the C=C–COOH fragment. This geometry favors π-electron delocalization across the aromatic ring and the unsaturated carboxylic side chain, which is consistent with the intense Raman bands observed in the 1550–1650 cm^−1^ region. These bands are mainly associated with aromatic and conjugated ν(C=C) stretching vibrations.

The optimized C=C bond lengths of the hydroxycinnamic side chain were very similar for p-coumaric, caffeic, and trans-ferulic acids, with values around 1.334–1.335 Å. This confirms that the conjugated vinylene fragment is preserved in the three molecules. Likewise, the carboxylic C=O bond lengths were nearly invariant, with values close to 1.203 Å. Therefore, the differences observed experimentally in the carbonyl and conjugated regions cannot be attributed to large structural distortions of the C=O or C=C bonds, but rather to differences in substituent effects, hydrogen-bonding ability, and electronic redistribution induced by hydroxylation and methoxylation.

The phenolic C–O distances showed small but chemically meaningful differences among the studied compounds. p-Coumaric acid presented one phenolic C–O bond of 1.355 Å, whereas caffeic acid showed two phenolic C–O bonds in the range of 1.350–1.368 Å. This broader range reflects the nonequivalent local environments of the catechol-type hydroxyl groups. Trans-ferulic acid showed a phenolic C–O bond of 1.349 Å and an additional aryl–O bond associated with the methoxy substituent of 1.362 Å. The O–CH_3_ bond length in the methoxy group was 1.412 Å. These structural features support the presence of methoxy-related vibrational contributions in the Raman spectrum of trans-ferulic acid, particularly in the fingerprint region where C–O stretching and aromatic deformation modes are expected.

Gallic acid displayed a different structural pattern because it lacks the vinylene C=C–COOH chain characteristic of hydroxycinnamic acids. Instead, the carboxylic group is directly attached to the aromatic ring, and the molecule contains three phenolic hydroxyl groups. The optimized phenolic C–O distances ranged from 1.353 to 1.367 Å, indicating nonequivalent hydroxyl environments around the hydroxybenzoic framework. This structural distinction explains why the Raman response of gallic acid differs from that of the hydroxycinnamic acids, particularly in the regions assigned to aromatic ring vibrations, C–O stretching, and O–H-related modes.

The optimized geometries indicate that the hydroxycinnamic acids share a common planar conjugated scaffold (Table 1). In contrast, gallic acid must be interpreted as a hydroxybenzoic system dominated by direct ring–carboxyl coupling and multiple phenolic hydroxyl groups. These geometric differences provide a molecular explanation for the spectral variations observed in the C=C, C=O, and C–O Raman regions.

Multiple rotamers may coexist, particularly for molecules containing several hydroxyl groups and rotatable carboxylic or methoxy fragments. Nevertheless, the selected conformers provide internally consistent reference structures for inspecting the normal modes used in the experimental band assignments.

### 2.4. DFT-Simulated Raman Spectra and Qualitative Comparison with Experiment

**Figure 4:**
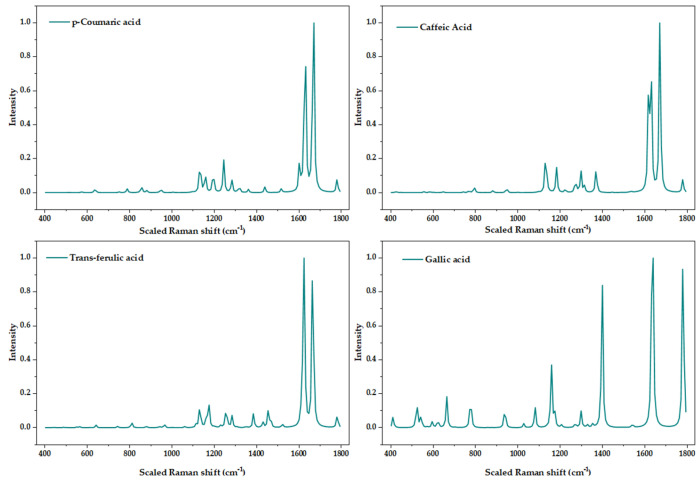
Normalized scaled DFT Raman spectra of p-coumaric, caffeic, trans-ferulic, and gallic acids calculated at the M06-2X/def2-TZVP level.

The normalized DFT-simulated Raman spectra of p-coumaric, caffeic, trans-ferulic, and gallic acids are shown in Figure 4. Qualitative comparison with the experimental solid-state band positions was performed using the frequencies and assignments summarized in Table 2. Because the calculations were conducted within the harmonic approximation for isolated molecules, the comparison focused on principal band positions and normal-mode character rather than on absolute Raman intensities.

The isolated-molecule harmonic model does not explicitly reproduce crystal packing, intermolecular hydrogen bonding, polymorphism, or the possible contribution of multiple conformers. Consequently, agreement with the solid-state spectra was evaluated qualitatively through the principal band positions and mode character. Periodic calculations, cluster models, or hydrogen-bonded dimers would be required for a more explicit description of the crystalline environment and would constitute relevant extensions for future work.

The simulated spectra reproduced the main experimental vibrational domains observed in the 400–1800 cm^−1^ region, supporting the suitability of the optimized geometries for vibrational interpretation. In particular, the calculated profiles captured the characteristic contributions associated with aromatic ring vibrations, ν(C=C) stretching modes, ν(C–O) vibrations, and carboxylic-group-related modes. This agreement indicates that the dominant Raman features of the studied phenolic acids are governed by their intrinsic molecular structure and can be rationalized by DFT-assisted normal-mode analysis.

For p-coumaric acid, the experimental spectrum was dominated by intense bands in the high-wavenumber region of the fingerprint window, especially around the aromatic/conjugated ν(C=C) domain. The theoretical spectrum reproduced this behavior, showing strong calculated Raman contributions in the same spectral region. Additional bands in the 1150–1300 cm^−1^ interval were observed in the simulated spectrum and can be assigned to coupled ν(C–O) and δ(O–H) modes, in-plane aromatic C–H deformation, and ring-vibrational modes. The overall correspondence confirms that the conjugated hydroxycinnamic framework mainly controls the Raman response of p-coumaric acid.

Caffeic acid showed a more complex vibrational pattern, consistent with the presence of two adjacent phenolic hydroxyl groups. The DFT spectrum reproduced the main experimental bands associated with the aromatic skeleton and the conjugated side chain, as well as several contributions in the C–O/O–H-sensitive region. The spectral complexity of caffeic acid arises from coupling between the catechol-type substituents and the aromatic π-system, which produces multiple vibrational contributions in the fingerprint region. Small deviations between calculated and experimental band positions are expected due to intermolecular interactions in the solid phase, particularly hydrogen bonding involving the phenolic hydroxyl groups.

For trans-ferulic acid, the theoretical spectrum showed strong agreement with the experimental profile in the regions associated with the substituted aromatic ring and the conjugated C=C–COOH side chain. The calculated spectrum reproduced the intense experimental bands above 1500 cm^−1^ and is mainly associated with aromatic and conjugated ν(C=C) stretching modes. In addition, the methoxy substituent introduces specific vibrational contributions involving C–O stretching and substituent-coupled ring deformations, which increase the spectral density of the 1100–1350 cm^−1^ region. Therefore, the comparison confirms that methoxylation modifies the Raman fingerprint by increasing the number of coupled vibrational modes involving the aromatic ring and oxygenated substituents.

Gallic acid exhibited a distinct experimental–theoretical relationship compared with the hydroxycinnamic acids. Although the principal calculated bands were consistent with the experimental spectral regions, the relative intensity distribution differed more noticeably. This behavior is chemically consistent with the hydroxybenzene character of gallic acid, which lacks the vinylene C=C–COOH fragment found in hydroxycinnamic structures. Consequently, its Raman response is dominated by the substituted aromatic ring, phenolic ν(C–O) vibrations, and carboxylic-group contributions. The presence of three phenolic hydroxyl groups also increases the possibility of intermolecular hydrogen bonding in the solid phase, which can affect band broadening and intensity redistribution relative to the isolated-molecule calculation.

The differences in relative intensity between experimental and theoretical spectra are not unexpected. Experimental Raman intensities in the solid state are influenced by crystal packing, molecular orientation, intermolecular hydrogen bonding, and local changes in polarizability within the condensed phase. Conversely, DFT-calculated Raman intensities correspond to isolated molecules and reflect changes in molecular polarizability under idealized harmonic conditions. Therefore, the most chemically meaningful criterion for validating the theoretical spectra is the reproduction of the principal band positions and the preservation of each molecule’s characteristic vibrational pattern.

Likewise, the gas-phase calculations were not intended to reproduce the ethanol/water spectra quantitatively. Implicit-solvation models such as PCM or SMD, or explicit solvent–solute clusters, could improve the description of solvent-dependent frequency shifts. In the present study, the solution spectra are therefore used only to identify qualitative environmental sensitivity.

The comparison between solid-state Raman spectra and DFT-simulated spectra indicates that the selected isolated-molecule model reproduces the principal vibrational regions sufficiently for qualitative mode assignment. The agreement in band positions supports the subsequent DFT-assisted assignment of the experimental Raman bands. The observed agreement supports the association of the principal Raman regions with the combined influence of oxygenated substituents, the conjugated hydroxycinnamic backbone, and coupled normal-mode displacements.

### 2.5. DFT-Assisted Vibrational Assignments

Building on the experimental–theoretical correspondence discussed in the previous section, the main Raman bands were assigned using the scaled DFT frequencies and the normal-mode character obtained from the optimized neutral structures. The resulting assignments are summarized in Table 2. Because phenolic acids contain several electronically coupled fragments, including the aromatic ring, carboxylic group, phenolic C–O bonds, O–H groups, and, in trans-ferulic acid, the methoxy substituent, most Raman bands should be interpreted as coupled vibrational modes rather than as fully localized bond vibrations.

The present study does not claim that previously reported assignments are generally incorrect. Rather, the calculated normal modes were used to refine and consolidate the interpretation of selected Raman bands by identifying the coupled participation of aromatic, carboxylic, phenolic, and methoxy-containing coordinates. Accordingly, the term “refined assignment” is used instead of “corrected assignment”.

The assignments confirm that the aromatic and conjugated framework primarily controls the high-wavenumber region of the fingerprint window. In the hydroxycinnamic acids, the bands located around 1600–1640 cm^−1^ were assigned to aromatic/conjugated ν(C=C) modes with contribution from the C=C–COOH side chain. The close agreement between the solid-state Raman bands and the scaled DFT frequencies in this region supports the interpretation that the hydroxycinnamic scaffold defines the main vibrational framework of p-coumaric, caffeic, and trans-ferulic acids.

The lower fingerprint region provided more specific information about each molecule’s substitution pattern. Bands between 1100 and 1350 cm^−1^ were assigned mainly to phenolic ν(C–O), δ(O–H), aromatic C–H in-plane deformation, and ring-coupled modes. Within this interval, caffeic acid showed contributions associated with its catechol-type hydroxyl arrangement, whereas trans-ferulic acid displayed additional methoxy-related ν(C–OCH_3_) character. In gallic acid, the same spectral region was dominated by the trihydroxylated aromatic motif, explaining its different profile relative to the hydroxycinnamic series.

The inclusion of solution Raman bands in Table 2 allows the environmental sensitivity of selected modes to be evaluated without replacing the solid-state spectra as the main reference for DFT comparison. Bands dominated by the aromatic/conjugated framework showed relatively small shifts between solid and solution, whereas modes involving ν(C=O), ν(C–O), and δ(O–H) were more affected by the solvent environment. This behavior is consistent with the direct participation of carboxylic and phenolic groups in hydrogen-bonding interactions with ethanol and water. Therefore, the assignments in Table 2 provide the basis for discussing hydroxylation-, methoxy-, and backbone-related trends within the selected molecular series.

### 2.6. Hydroxylation-Related Trends Within the Selected Molecular Series

Because the molecular series does not independently vary only the number of hydroxyl groups, the following comparison should not be interpreted as a strict isolation of the hydroxylation effect. The most direct comparison is between p-coumaric and caffeic acids, which share the hydroxycinnamic backbone and differ by one additional phenolic hydroxyl group. Gallic acid is discussed separately because it also differs in molecular backbone. The assignments in Table 2 indicate that hydroxylation mainly affects the Raman response in the 1100–1350 cm^−1^ region, where phenolic ν(C–O), δ(O–H), and ring-coupled modes contribute significantly.

In p-coumaric acid, the single phenolic hydroxyl group produces a relatively defined set of bands in the C–O/O–H-sensitive region. The bands at 1168.0 and 1256.7 cm^−1^, supported by scaled DFT frequencies at 1158.1 and 1244.9 cm^−1^, indicate that the phenolic group contributes to the fingerprint region through coupled C–O stretching, O–H deformation, and aromatic ring motion. The limited number of hydroxyl-related contributions explains the comparatively simpler spectral pattern of p-coumaric acid.

Caffeic acid shows a more congested vibrational response in the same region due to the catechol arrangement. The bands at 1181.5 and 1297.2 cm^−1^ were assigned to C–O/O–H-coupled modes, and their DFT counterparts confirm the participation of the adjacent phenolic groups. The proximity of the two hydroxyl substituents increases vibrational coupling within the aromatic ring and enhances the sensitivity of these bands to the local environment. This is reflected in the larger solid-to-solution displacement of the catechol-associated bands compared with the more structurally isolated phenolic mode in p-coumaric acid.

Gallic acid provides the most hydroxylated case and therefore the clearest example of how multiple phenolic groups reshape the Raman fingerprint. Its band at 1300.1 cm^−1^, assigned to phenolic ν(C–O) and δ(O–H) contributions, agrees closely with the scaled DFT frequency at 1299.9 cm^−1^. The additional band at 1137.2 cm^−1^, assigned to a ring + ν(C–O) coupled mode, further confirms that the trihydroxylated aromatic framework dominates the lower fingerprint region. The larger shifts observed for these bands in solution indicate that the three hydroxyl groups strongly increase the molecule’s ability to interact via hydrogen bonding.

Thus, hydroxylation controls both the number and the environmental sensitivity of Raman-active modes in the fingerprint region. The progression from p-coumaric acid to caffeic acid shows how a catechol unit increases vibrational coupling within the hydroxycinnamic scaffold. In contrast, gallic acid demonstrates how a trihydroxylated hydroxybenzoic framework generates a distinct C–O/O–H-dominated spectral response.

### 2.7. Methoxy-Related Vibrational Features of Trans-Ferulic Acid

Trans-ferulic acid provides evidence of methoxy-associated vibrational contributions; however, the comparison does not isolate methoxylation as the only structural variable because the substitution pattern also differs from those of p-coumaric and caffeic acids. The discussion is therefore restricted to spectral features supported by the calculated normal modes involving the aryl–OCH_3_ fragment.

The methoxy-sensitive region is mainly located between 1200 and 1300 cm^−1^, where trans-ferulic acid shows a band at 1271.2 cm^−1^ in the solid state and a scaled DFT counterpart at 1256.4 cm^−1^. This band was assigned to a mixed ν(C–OCH_3_) + ν(C–O) contribution, indicating that the methoxy group is not spectroscopically isolated but coupled with the phenolic C–O bond and the aromatic framework. The presence of this contribution differentiates trans-ferulic acid from p-coumaric acid, whose analogous region is governed mainly by phenolic C–O/O–H modes.

Methoxylation also modifies the ring deformation region. The band at 1430.3 cm^−1^, reproduced by the scaled DFT frequency at 1429.7 cm^−1^, was assigned to an aromatic ring/methoxy-coupled deformation. This mode shows that the –OCH_3_ group influences not only local C–O vibrations but also the collective motion of the substituted aromatic ring. Compared with caffeic acid, where hydroxyl-related and ring-coupled contributions dominate the same region, trans-ferulic acid exhibits a different balance between donor substituent effects and methoxy-induced polarizability changes.

The spectral behavior of trans-ferulic acid is therefore not merely the result of adding a new substituent vibration. The methoxy group modifies the Raman fingerprint by increasing the number of oxygenated-substituent modes and by altering the polarizability response of the aromatic π-system. This explains why trans-ferulic acid shows a more structured fingerprint region while maintaining the characteristic hydroxycinnamic bands above 1500 cm^−1^.

Consequently, methoxylation acts as a dual structural modifier: it introduces specific ν(C–OCH_3_)-related vibrations and perturbs the ring modes through electronic coupling with the aromatic system. This effect is distinct from hydroxylation because the methoxy group does not behave as a hydrogen-bond donor. However, it still contributes strongly to the Raman response through C–O stretching and polarizability modulation.

### 2.8. Comparative Influence of the Molecular Backbone and π-Conjugated Side Chain

The comparison between the three hydroxycinnamic acids and gallic acid simultaneously involves changes in conjugation, side-chain topology, and hydroxylation pattern. Therefore, the observed differences are attributed to the combined molecular-backbone effect rather than exclusively to π-conjugation. The previous sections show that hydroxylation and methoxylation modulate specific regions of the Raman fingerprint. However, the global spectral organization is primarily determined by the molecular backbone. This effect becomes evident when the hydroxycinnamic acids, p-coumaric, caffeic, and trans-ferulic acids, are compared with gallic acid. The former contain a conjugated C=C–COOH side chain, whereas gallic acid is a hydroxybenzoic acid with the carboxylic group directly attached to the aromatic ring.

The 1580–1640 cm^−1^ region is the most representative marker of the hydroxycinnamic backbone. In p-coumaric, caffeic, and trans-ferulic acids, the bands at 1603.9, 1611.6, and 1601.0 cm^−1^, respectively, were assigned to aromatic/conjugated ν(C=C) modes. The corresponding DFT-scaled frequencies support these bands and reflect the extended delocalization between the aromatic ring and the C=C–COOH side chain. The persistence of this band family across the three hydroxycinnamic acids indicates that the conjugated side chain establishes a common vibrational framework.

The second high-wavenumber contribution, assigned to coupled ν(C=C)/ν(C=O) modes, further reflects the connection between the vinylene bridge and the carboxylic group. In p-coumaric and trans-ferulic acids, the corresponding experimental bands at 1634.7 and 1628.9 cm^−1^ are closely reproduced by the scaled DFT frequencies. This agreement supports the interpretation that the carboxylic fragment in hydroxycinnamic acids does not vibrate independently but participates in modes coupled with the conjugated π-system.

Gallic acid deviates from this pattern because it lacks the vinylene bridge. Its Raman response is instead governed by the hydroxybenzoic aromatic framework, direct ring–carboxyl coupling, and multiple contributions from phenolic C–O/O–H bonds. The carbonyl-related band at 1739.8 cm^−1^ and the ring-associated band at 1668.5 cm^−1^ illustrate this different coupling scheme. Unlike the hydroxycinnamic acids, where the C=C–COOH side chain contributes strongly to the Raman response, gallic acid distributes its vibrational intensity among the carboxylic group, substituted ring, and phenolic oxygenated modes.

Therefore, the conjugated hydroxycinnamic backbone is associated with a common high-wavenumber vibrational pattern, whereas hydroxyl and methoxy substituents modulate the detailed fingerprint. The contrast with gallic acid demonstrates the combined influence of the molecular backbone, side-chain topology, and substitution pattern. It should not be interpreted as an isolated measure of the π-conjugation effect.

### 2.9. Molecular Electrostatic Potential Analysis

**Figure 5:**
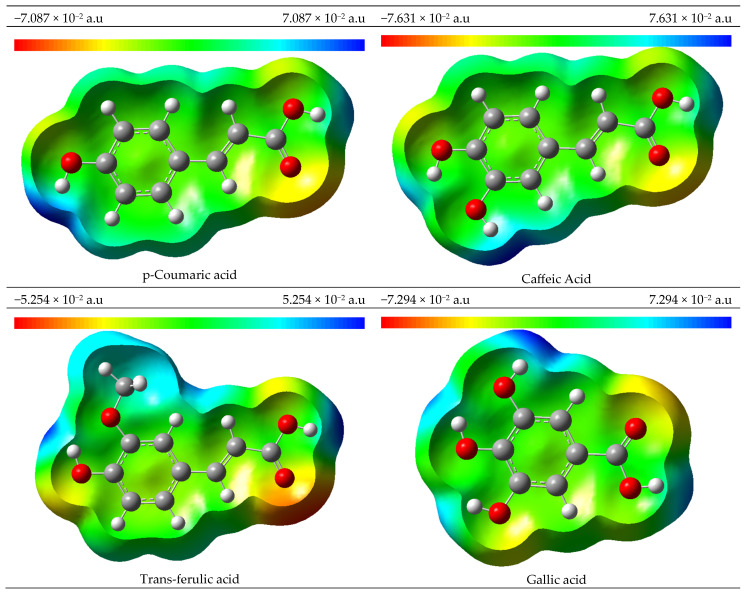
Molecular electrostatic potential maps of p-coumaric, caffeic, trans-ferulic, and gallic acids calculated from the optimized DFT structures.

To complement the vibrational interpretation, molecular electrostatic potential (MEP) maps were calculated for the optimized structures of p-coumaric, caffeic, trans-ferulic, and gallic acids (Figure 5). This analysis provides a spatial representation of the electron-rich and electron-deficient regions of each molecule, thereby rationalizing the Raman shifts associated with solvent-sensitive functional groups from an electrostatic perspective. According to the color scale used in the maps, negative electrostatic potential is mainly localized around oxygen atoms. In contrast, positive potential is concentrated near acidic hydrogen atoms, particularly those belonging to phenolic and carboxylic O–H groups.

For all phenolic acids, the most negative regions were associated with the oxygen atoms of the carboxylic and phenolic groups. These electron-rich sites are expected to act as hydrogen-bond acceptor regions and are directly related to the Raman bands assigned to ν(C=O), ν(C–O), and coupled C–O/O–H modes. Therefore, the MEP distribution supports the interpretation that the vibrational modes involving oxygenated functional groups are more sensitive to changes in the molecular environment than modes dominated by the aromatic carbon framework.

In p-coumaric acid, the negative potential is concentrated around the phenolic oxygen and the carboxylic group, while the aromatic and conjugated carbon framework shows a more neutral electrostatic distribution. This agrees with the Raman behavior discussed above: the conjugated ν(C=C)-dominated bands remain comparatively stable, whereas the bands involving C–O, O–H, and C=O coordinates are more susceptible to environmental perturbation.

Caffeic acid exhibits a broader negative electrostatic region over the catechol fragment due to the presence of two adjacent phenolic hydroxyl groups. This distribution increases the number of potential interaction sites and explains the higher sensitivity of its C–O/O–H-associated Raman bands. The MEP map therefore provides an electrostatic basis for the greater spectral complexity observed in the hydroxylation-sensitive fingerprint region.

In trans-ferulic acid, negative potential is distributed over the phenolic oxygen, the methoxy oxygen, and the carboxylic group. The methoxy substituent introduces an additional electron-rich site, although it does not provide an O–H proton that can act as a hydrogen-bond donor. This distinction is important because the methoxy group modifies the local electrostatic and polarizability environment of the aromatic ring, contributing to the Raman bands assigned to ν(C–OCH_3_) and methoxy-coupled ring modes without behaving in the same way as a phenolic hydroxyl group.

Gallic acid shows the most extensive oxygen-rich electrostatic surface among the studied compounds, consistent with the presence of three phenolic hydroxyl groups and one carboxylic acid group. The accumulation of negative potential around these oxygenated sites explains why its Raman response is dominated by C–O/O–H and carboxylic contributions rather than by an extended conjugated C=C–COOH framework. This electrostatic pattern also supports the strong environmental sensitivity of the bands assigned to phenolic C–O and carbonyl-related vibrations.

The positive electrostatic regions located near the O–H protons further confirm the hydrogen-bond donor character of the phenolic and carboxylic groups. Consequently, the MEP maps explain why bands involving O–H bending, C–O stretching, and C=O stretching are more affected by intermolecular interactions and solvent exposure. In contrast, the aromatic and conjugated carbon skeletons display less pronounced electrostatic extrema, which is consistent with the comparatively higher stability of the Raman bands assigned to aromatic/conjugated ν(C=C) modes.

The MEP analysis links the electronic surface properties of the phenolic acids with their vibrational behavior. The electrostatic concentration over oxygenated functional groups provides a molecular explanation for the solvent sensitivity of C–O, O–H, and C=O Raman bands, while the less polar aromatic/conjugated regions account for the relative robustness of the ν(C=C)-dominated bands. Thus, the MEP maps reinforce the structure–vibrational relationship established from the Raman and DFT assignments.

### 2.10. HOMO–LUMO Analysis and Electronic Descriptors

**Figure 6:**
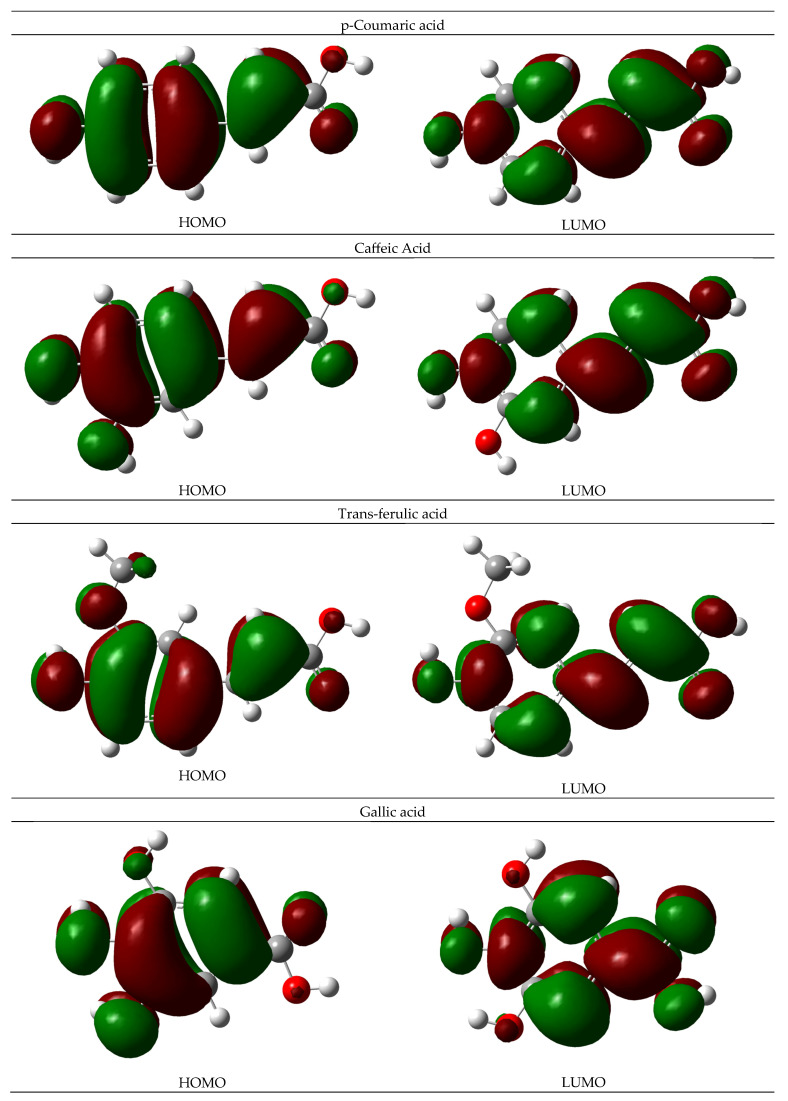
HOMO and LUMO distributions of p-coumaric, caffeic, trans-ferulic, and gallic acids obtained from the optimized DFT structures.

After identifying the electrostatic regions associated with the oxygenated functional groups, the frontier molecular orbitals were analyzed to assess how electronic delocalization contributes to the Raman response of the phenolic acids studied. The HOMO and LUMO distributions are shown in Figure 6, and the corresponding frontier orbital energies and electronic descriptors are summarized in Table 3.

For the hydroxycinnamic acids, p-coumaric, caffeic, and trans-ferulic acids, the frontier orbitals were mainly distributed over the aromatic ring and the conjugated C=C–COOH side chain. This confirms that the vinylene bridge promotes electronic communication between the aromatic π-system and the carboxylic fragment. Such delocalization is consistent with the intense Raman bands previously assigned to aromatic/conjugated ν(C=C) modes and to coupled ν(C=C)/ν(C=O) vibrations. The HOMO–LUMO distributions provide an electronic basis for the characteristic high-wavenumber vibrational pattern of the hydroxycinnamic backbone.

Among the hydroxycinnamic acids, trans-ferulic acid showed the highest HOMO energy and the smallest HOMO–LUMO gap. This behavior is consistent with the electron-donating character of the methoxy substituent, which increases the aromatic system’s electronic richness and facilitates changes in polarizability during vibration. Trans-ferulic acid exhibited the highest calculated isotropic polarizability among the studied molecules. Trans-ferulic acid exhibited the highest calculated isotropic polarizability among the selected molecules, indicating greater overall electronic deformability. However, static isotropic polarizability should not be directly equated with experimental Raman intensity, because the Raman activity of a normal mode depends on the derivative of the polarizability tensor with respect to the corresponding normal coordinate. Accordingly, α_iso_ is discussed here only as a complementary electronic descriptor.

Caffeic acid presented an intermediate polarizability and a slightly larger energy gap than trans-ferulic acid. The HOMO distribution was influenced by the catechol-type hydroxyl arrangement, indicating that the adjacent phenolic groups participate in the frontier electronic structure. However, the electronic effect of hydroxylation differs from that of methoxylation: hydroxyl groups strongly affect local electrostatic interactions and hydrogen-bonding sensitivity, whereas the methoxy group more clearly enhances the polarizable character of the aromatic framework.

Gallic acid displayed the largest HOMO–LUMO gap and the lowest isotropic polarizability. This result is consistent with its hydroxybenzoic structure, which lacks the extended C=C–COOH side chain found in hydroxycinnamic acids. Its frontier orbitals were mainly confined to the substituted aromatic ring and the directly attached carboxylic group, indicating a more localized electronic structure. This reduced delocalization helps explain why gallic acid exhibited a Raman profile distinct from that of the hydroxycinnamic series, with a smaller contribution from extended conjugated stretching modes and a greater influence from phenolic C–O/O–H and ring-localized vibrations.

The dipole moments also reflected differences in molecular asymmetry and substituent distribution. Trans-ferulic acid showed the highest dipole moment, followed by p-coumaric, gallic, and caffeic acids. The larger dipole moment of trans-ferulic acid can be related to the combined orientation of the methoxy, phenolic, and carboxylic groups along the conjugated molecular framework. Although the dipole moment is not directly proportional to Raman intensity, it provides complementary information about charge separation and molecular polarity, which can influence intermolecular interactions and vibrational sensitivity.

The HOMO–LUMO analysis indicates that the Raman behavior of the studied phenolic acids is not controlled only by the presence of specific functional groups, but also by the extent of electronic delocalization and molecular polarizability. The hydroxycinnamic acids share a delocalized frontier-orbital pattern associated with the conjugated C=C–COOH backbone, whereas gallic acid shows a more localized hydroxybenzoic electronic structure. These results reinforce the interpretation that π-conjugation and substituent-driven changes in polarizability are central factors governing the Raman response of phenolic acids.

### 2.11. LOL Analysis and Electron Localization

**Figure 7:**
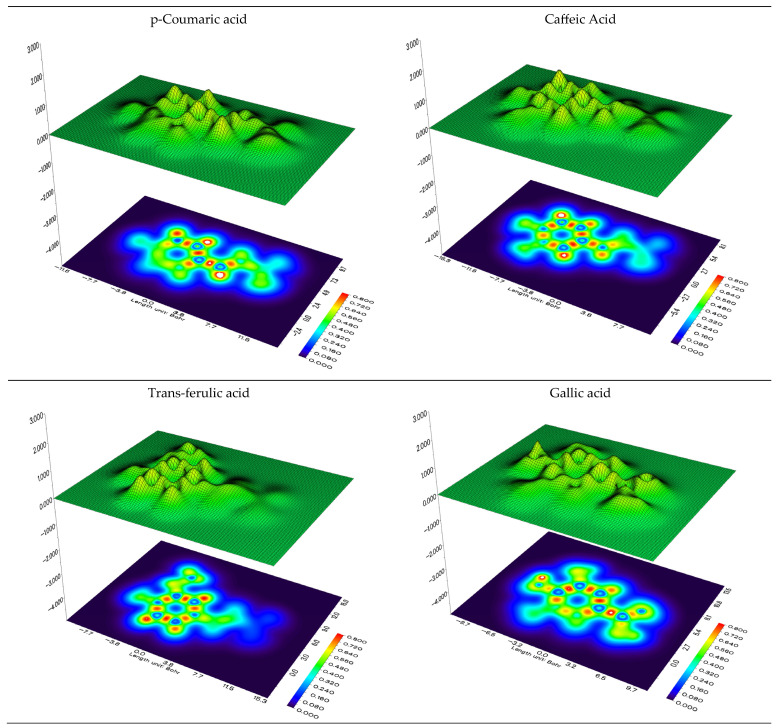
LOL maps of p-coumaric, caffeic, trans-ferulic, and gallic acids calculated from the optimized DFT structures.

The localized orbital locator is a dimensionless real-space descriptor derived from local electronic kinetic energy density terms. High LOL values identify regions of comparatively strong electron localization, such as covalent-bonding and lone-pair domains, whereas lower values indicate more delocalized electronic regions. Unlike the molecular electrostatic potential, which describes the electrostatic interaction energy of a positive test charge, LOL characterizes the spatial localization of electrons. Therefore, the two analyses provide complementary but physically distinct information. The LOL maps are shown in Figure 7. While the MEP analysis identified the electrostatic distribution over the molecular surface and the HOMO–LUMO analysis described frontier-orbital delocalization, the LOL analysis provides complementary information on the spatial localization of electron density associated with bonds and lone-pair regions. This is particularly relevant for interpreting Raman bands involving oxygenated groups, since C–O, C=O, and O–H vibrations are strongly influenced by the local electronic organization around oxygen atoms.

In all molecules, high LOL domains were observed around the oxygen-containing functional groups, reflecting the localized character of oxygen lone pairs and σ-bonding regions. These localized regions are associated with phenolic C–O bonds, carboxylic C=O and C–OH bonds, and O–H groups. The presence of localized electron density around these sites supports the vibrational assignments previously attributed to ν(C–O), ν(C=O), δ(O–H), and coupled C–O/O–H modes. Therefore, the LOL maps reinforce the interpretation that oxygenated substituents are not only electrostatically active, as shown by the MEP maps, but also electronically localized in a way that strongly modulates the Raman fingerprint.

For p-coumaric acid, the LOL distribution highlights the phenolic hydroxyl group and the carboxylic acid fragment as the principal localized electronic regions outside the aromatic π-system. This pattern is consistent with its comparatively simpler Raman response in the C–O/O–H-sensitive region, since only one phenolic hydroxyl group contributes significantly to these localized vibrational coordinates. The conjugated aromatic side-chain region, in contrast, exhibits a more delocalized electronic character, consistent with the intense ν(C=C)-dominated bands discussed in the previous sections.

Caffeic acid shows a more pronounced localization pattern around the catechol fragment. The two adjacent phenolic hydroxyl groups generate multiple localized regions of electron density associated with neighboring C–O and O–H coordinates. This electronic arrangement explains the higher density of bands assigned to C–O/O–H-coupled modes and the greater sensitivity of these bands to the molecular environment. The catechol unit therefore acts as a localized electronic domain superimposed on the hydroxycinnamic π-framework, producing a more complex fingerprint region than that observed for p-coumaric acid.

In trans-ferulic acid, the LOL map differentiates the phenolic hydroxyl group from the methoxy substituent. The methoxy oxygen contributes a localized electron-rich region associated with the aryl–OCH_3_ fragment, whereas the phenolic oxygen contributes both C–O and O–H-related localization. This distinction supports the previous assignment of bands involving mixed ν(C–OCH_3_) and phenolic ν(C–O) character. The methoxy group therefore modifies the Raman response through localized C–O electronic structure and through its coupling with the aromatic ring, without contributing an O–H donor site.

Gallic acid exhibits the most extensive oxygen-centered localization pattern among the studied molecules. The three phenolic hydroxyl groups and the carboxylic acid group generate multiple localized regions around C–O, O–H, and C=O coordinates. This explains why the Raman response of gallic acid is dominated by phenolic C–O/O–H and carboxylic contributions rather than by an extended hydroxycinnamic conjugated chain. The LOL map therefore supports the interpretation of gallic acid as a trihydroxylated hydroxybenzoic system with strong local electronic contributions from oxygenated groups.

The connection between LOL and the solid-to-solution Raman behavior is especially relevant for oxygen-centered modes. The regions of higher electron localization around phenolic and carboxylic oxygen atoms coincide with the sites most likely to interact with ethanol and water through hydrogen bonding. As a result, vibrational modes involving ν(C–O), ν(C=O), and δ(O–H) are more prone to shifts, broadening, or intensity redistribution when the molecular environment changes from the solid state to solution. In contrast, modes dominated by the aromatic/conjugated carbon framework are less directly exposed to localized solvent interactions and therefore tend to show greater spectral stability.

### 2.12. Chemometric Discrimination of Solid and Solution Raman Spectra

**Figure 8:**
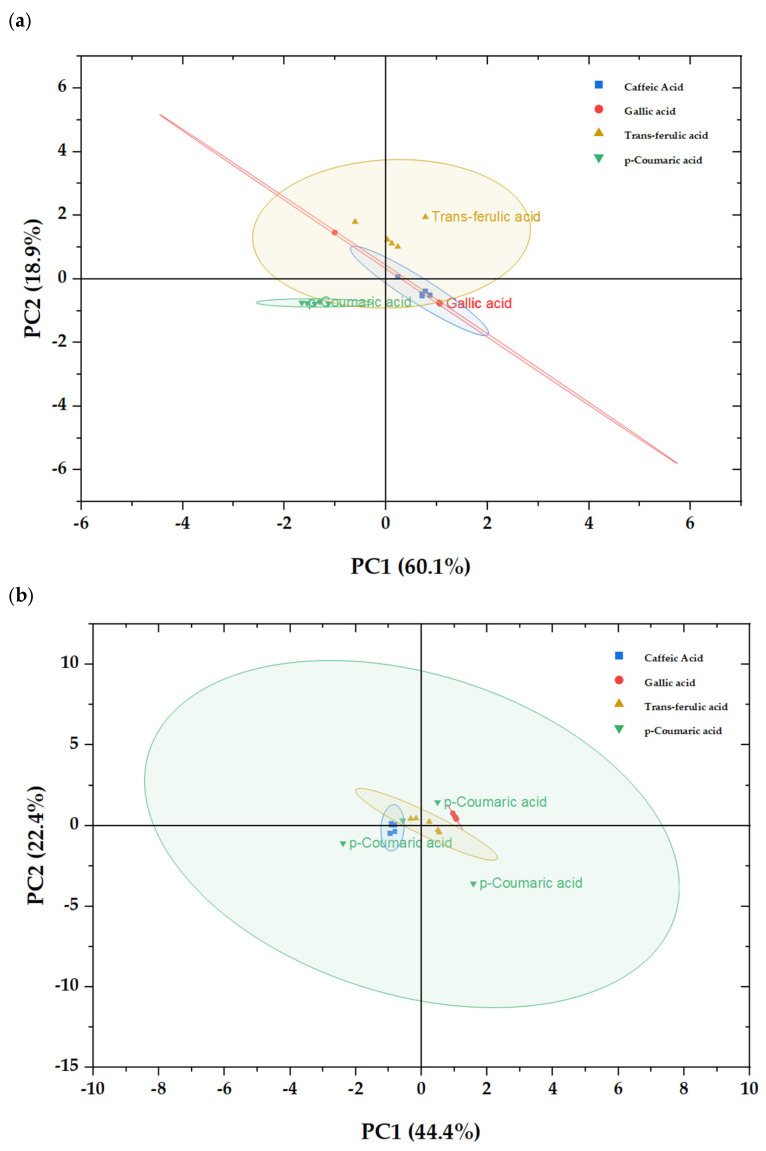
PC1–PC2 score plots obtained from the solid-state and solution Raman datasets: (**a**) solid-state spectra and (**b**) solution spectra. Each point represents an individual replicate spectrum. The plots are presented to explore spectral similarities, differences, and within-group dispersion visually.

PCA was used as an exploratory visualization tool rather than as a validated classification model. Five replicate spectra were included for each compound under each physical condition, yielding 20 observations for the solid-state model and 20 for the solution model. Principal component analysis was applied separately to the solid-state and solution Raman spectra to evaluate whether the vibrational fingerprints were sufficient to distinguish the studied phenolic acids under each physical condition. This strategy avoids forcing solid and solution spectra into a single multivariate space, where the first principal component could be dominated by the physical state rather than by molecular differences. The resulting PCA score plots are shown in Figure 8.

For the solid-state Raman spectra, PC1 and PC2 explained 60.1% and 18.9% of the total variance, respectively, accounting for 79.0% of the spectral variability in the first two components. This PCA model showed the clearest structural discrimination among the studied compounds. Trans-ferulic acid was mainly separated along the positive PC2 direction, whereas gallic acid was displaced toward positive PC1 and negative PC2. p-Coumaric acid occupied a more negative PC1 region, while caffeic acid appeared closer to the central region of the score space. This distribution indicates that the solid-state Raman spectra retain sufficient molecular information to differentiate the phenolic acids by their substitution patterns and molecular backbones.

The separation observed in the solid-state PCA is consistent with the vibrational assignments discussed above. The hydroxycinnamic acids share bands associated with the conjugated aromatic C=C–COOH framework, but their score positions are modulated by substituent-specific contributions. Trans-ferulic acid differs from the non-methoxylated hydroxycinnamic acids due to methoxy-related C–O and ring-coupled vibrations, particularly in the fingerprint region. Gallic acid, in contrast, is structurally distinct because it lacks the vinylene C=C–COOH chain and contains a trihydroxylated hydroxybenzoic framework. Its displacement in the PCA space therefore reflects a different balance between aromatic ring modes, phenolic C–O/O–H vibrations, and carboxylic-group contributions.

The PCA model derived from the solution spectra exhibited different behavior. In this case, PC1 and PC2 explained 44.4% and 22.4% of the total variance, respectively, for a total of 66.8% of the accumulated variance. Compared with the solid-state PCA, the separation among compounds was less defined, and the score clusters were more compressed around the center of the plot. This behavior indicates that solvation reduces the spectral contrast among the phenolic acids, most likely through band broadening, solvent contribution, and hydrogen-bonding interactions with the oxygenated functional groups.

The broader dispersion observed for some solution spectra suggests that the Raman response in solution is more sensitive to the local molecular environment than the solid-state response. This effect is particularly relevant for bands involving ν(C=O), ν(C–O), and δ(O–H), since carboxylic and phenolic groups can interact with ethanol and water. Therefore, while the solution spectra still contain molecularly relevant information, their discriminative power is partially diminished by solvent-induced spectral modulation.

Taken together, the two PCA models demonstrate that the solid-state Raman spectra provide the most robust chemometric discrimination of the studied phenolic acids. The solution spectra retain part of the molecular fingerprint but show lower class separation due to environmental effects. This comparison supports the use of solid-state Raman spectra as the primary dataset for structure–vibrational correlation, while the solution spectra provide complementary information about the sensitivity of oxygenated vibrational modes to solvent interactions. The position of gallic acid in the solid-state score plot is consistent with its distinct hydroxybenzoic backbone; however, the within-group dispersion may also reflect heterogeneity in packing or sampling position. The broader distribution of p-coumaric acid in solution may arise from the lower spectral contrast and greater relative contribution of the ethanol/water background. These explanations remain tentative because no independent instrumental or physicochemical variable was measured to isolate the origin of the dispersion.

The dispersion of the score values should not be interpreted exclusively as an intrinsic molecular property. In the solid-state dataset, variations may also reflect local sample heterogeneity, focusing differences, and residual baseline variation. In the solution dataset, solvent contributions, lower signal-to-background ratios, and differences in band broadening may account for the greater dispersion observed in some replicate spectra. Given the limited number of replicates and the absence of external validation, the PCA results are considered exploratory.

Taken together, the results indicate that recognizable functional-group coordinates define the principal Raman domains, while vibrational coupling, backbone structure, substitution pattern, and molecular environment modulate their detailed spectral expression. The conclusions are restricted to the four selected phenolic acids and to the single-conformer isolated-molecule models used in the calculations.

## 3. Materials and Methods

### 3.1. Reagents

The phenolic compounds selected for this study were p-coumaric acid, caffeic acid, trans-ferulic acid, and gallic acid. p-Coumaric acid with 98% purity, caffeic acid with 95% purity, trans-ferulic acid with 99% purity, and gallic acid with 97.5% purity were used. Furthermore, 99% ethanol was used as an auxiliary solvent for solution preparation when required. All samples were handled in the dark and stored in closed containers to minimize oxidation or moisture absorption, following general precautions recommended for phenolic-rich materials and plant-derived bioactive compounds [25,26].

Before Raman analysis, the solid samples were visually inspected to discard evident color changes, agglomeration, or physical contamination. Subsequently, the samples were dried at 40–45 °C for 12–24 h and stored in closed vials until spectroscopic analysis.

### 3.2. Molecular Design of the Study

The study was designed as a comparative analysis of four naturally occurring oxygenated phenolic acids with distinct substitution patterns and molecular backbones. p-Coumaric, caffeic, and trans-ferulic acids belong to the hydroxycinnamic family and share the conjugated Ar–CH=CH–COOH framework. Within this subset, p-coumaric acid contains one phenolic hydroxyl group, caffeic acid contains a catechol-type dihydroxylated ring, and trans-ferulic acid contains both phenolic hydroxyl and methoxy substituents. These three compounds therefore provide a common hydroxycinnamic platform for examining substitution-related differences in the Raman fingerprint [27,28].

Gallic acid was included as a hydroxybenzoic structural contrast because it contains three phenolic hydroxyl groups but lacks the conjugated vinyl side chain characteristic of hydroxycinnamic acids. Its inclusion enables comparison between a highly hydroxylated hydroxybenzoic framework and the conjugated hydroxycinnamic systems. However, because gallic acid differs simultaneously in substitution pattern and molecular backbone, it was not treated as a direct reference for isolating the specific effect of π-conjugation.

Cinnamic acid, which contains the same Ar–CH=CH–COOH framework without phenolic hydroxyl or methoxy substituents, would provide an appropriate unsubstituted reference for a more rigorous isolation of aromatic-substitution effects. Because cinnamic acid was not part of the original experimental design, the present study is restricted to comparative structure–vibrational trends among the four selected phenolic acids. It does not claim to independently quantify hydroxylation, methoxylation, or π-conjugation effects. This selection enabled a qualitative comparison of hydroxyl-, methoxy-, and molecular-backbone-related trends within the investigated molecular set.

### 3.3. Solid-State Raman Analysis

Experimental Raman spectra were first recorded in the solid state to obtain the direct vibrational response of each compound without significant interference from solvent, ionization, or solution aggregation effects. For each sample, a small amount of the dry solid was deposited on a glass slide or a silicon support. The powder was homogeneously distributed and gently compacted to favor microscopic focusing and improve spectral reproducibility [29].

For each compound, at least five spectra were acquired at different points of the sample to evaluate signal homogeneity. The main spectral range considered was 400–1800 cm^−1^, because this region contains the most relevant vibrations of the aromatic ring, carboxyl group, and C–O/O–H bonds. Additionally, when the signal allowed it, the 2800–3600 cm^−1^ region was recorded to observe C–H and O–H vibrations [30,31].

The term “optimized experimental conditions” refers exclusively to the instrumental parameters reported in Table 4. These conditions were applied consistently to the studied compounds. They were selected to obtain an adequate signal-to-background ratio while avoiding detector saturation, visible photothermal degradation, or spectral changes during consecutive acquisitions. Five replicate spectra were recorded at different sampling positions for each solid sample and from independent acquisitions of each solution. The spectra acquired between 50 and 3500 cm^−1^ were subsequently cropped to the 400–1800 cm^−1^ region for vibrational interpretation and principal component analysis.

### 3.4. Raman Analysis in Solution

As a complementary analysis, solutions of the phenolic acids were prepared to evaluate the effect of the medium on the vibrational response. An ethanol/water mixture (1:1, *v*/*v*) was used as the solvent for all solution measurements. When the solubility of any compound was limited, dissolution was facilitated by gentle stirring or mild sonication, with avoidance of prolonged heating [32].

The concentration considered was 1 × 10^−2^ M. The Raman spectrum of the solvent blank was also recorded under the same instrumental conditions to identify potential contributions from the medium. The solution spectra were used only as complementary comparative information, whereas the main vibrational assignment was based on the solid-state spectra and DFT calculations [33].

### 3.5. Raman Spectral Preprocessing

All Raman spectra were processed using an identical and sequential preprocessing workflow to ensure consistency between compounds and physical states. The raw spectra were initially cropped to the 400–1800 cm^−1^ region, which contains the principal fingerprint bands associated with the aromatic ring, carboxylic group, phenolic C–O/O–H coordinates, and methoxy-related vibrations. Baseline correction was subsequently performed using an asymmetric least-squares algorithm with a smoothing parameter of λ = 1 × 10^5^, an asymmetry parameter of *p* = 0.001, and 10 iterative cycles to reduce fluorescence contributions and remove background-slope effects. Spectral smoothing was then applied using a Savitzky–Golay filter with a second-order polynomial and an 11-point window. The same preprocessing parameters were applied to all spectra to reduce instrumental noise without producing detectable changes in band position, width, or overall spectral profile [34,35].

For visual comparison and construction of the representative Raman profiles, each spectrum was normalized to its maximum intensity within the selected spectral interval. This procedure was used exclusively to compare band positions and relative spectral distributions and was not interpreted as a measure of absolute Raman scattering efficiency among the compounds. For principal component analysis, the individual replicate spectra were vector-normalized and subsequently mean-centered, as described in Section 3.9. No additional autoscaling was applied.

Five replicate spectra were retained as independent observations for each phenolic acid and physical state in the multivariate analysis. The replicate spectra were not averaged before PCA. Mean spectra were calculated only for graphical representation and comparative vibrational assignment. Spectral repeatability was evaluated by examining the consistency of the principal band positions and the overall spectral profiles across the five replicate measurements. The ethanol/water blank spectrum was subjected to the same preprocessing procedure and was used to identify spectral regions potentially affected by solvent contributions; however, it was not included as an observation in the PCA models [36,37].

### 3.6. Computational DFT Calculations

The molecular structures of p-coumaric, caffeic, trans-ferulic, and gallic acids were built in their neutral form. For the hydroxycinnamic acids, the trans configuration of the C=C–COOH chain was considered because this arrangement is structurally relevant for preserving conjugation between the aromatic ring and the carboxyvinyl fragment [20]. One conformer was selected for each neutral molecule and optimized in the gas phase. An exhaustive conformational search was not performed. Therefore, the calculated spectra represent the harmonic vibrational response of the selected isolated structures and do not constitute Boltzmann-weighted conformational ensembles.

The initial geometries were preoptimized using a molecular force field and subsequently optimized by density functional theory using Gaussian 16 B.01. Geometry optimizations and Raman vibrational frequency calculations were performed using the hybrid meta-GGA M06-2X functional in combination with the def2-TZVP basis set. This level of theory was selected because it provides an adequate balance between computational cost and the description of structural, electronic, and vibrational properties of oxygenated aromatic systems [38,39]. All calculations were carried out for neutral molecules in the singlet state.

After each geometry optimization, vibrational frequency calculations were performed at the same M06-2X/def2-TZVP level to confirm the stationary nature of the optimized structures. A uniform scaling factor of 0.967 was applied consistently to all calculated harmonic frequencies to facilitate comparison with experiment. This empirical correction reduces the systematic overestimation associated with the harmonic approximation; however, it does not account for conformational, crystal-packing, or solvent effects [40,41].

The theoretical Raman spectra were generated using Gaussian broadening with a full width at half maximum of 10 cm^−1^. The simulated intensities were normalized to facilitate qualitative comparison with the experimental Raman spectra, prioritizing agreement in band positions over direct comparison of absolute intensities [42]. Crystal-packing interactions, intermolecular hydrogen bonds, and explicit ethanol/water interactions were not included in the computational models. The theoretical spectra were therefore used for qualitative normal-mode assignment and comparison of principal band positions, rather than for exact reproduction of condensed-phase spectra.

### 3.7. Molecular Electrostatic Potential, Frontier Orbitals, and LOL Analysis

From the optimized geometries, molecular electrostatic potential (MEP) maps were calculated and visualized to identify the distribution of electrophilic and nucleophilic regions in each phenolic acid [43]. This analysis enabled the identification of negative-potential zones, mainly associated with the oxygen atoms of carboxyl, hydroxyl, and methoxy groups, as well as positive-potential regions related to acidic protons or sites susceptible to hydrogen-bonding interactions.

The HOMO and LUMO frontier molecular orbitals were analyzed to assess the distributions of donor and acceptor electron densities, the extent of π-delocalization, and the effects of hydroxylation, methoxylation, and conjugation on the compounds’ electronic structure. For each molecule, EHOMO and ELUMO values were reported, and the energy gap ΔE = ELUMO − EHOMO was used as a comparative descriptor of electronic stability and relative molecular reactivity [44,45].

Additionally, localized orbital locator (LOL) analysis was performed to examine electron localization in covalent bonds, lone-pair regions, and domains associated with oxygenated substituents. LOL representations complemented the interpretation of MEP and frontier orbitals, particularly for identifying regions of high electron localization around phenolic, methoxy, and carboxylic groups [46,47]. MEP, HOMO, LUMO, and LOL surfaces were generated from the wavefunction files obtained from the DFT calculations using molecular visualization tools such as GaussView 6.0.16 and Multiwfn 3.8 [48].

### 3.8. Vibrational Assignment

Raman band assignment was performed by comparing experimental frequencies with scaled DFT frequencies. For each relevant band, the corresponding normal mode was inspected to identify the dominant contribution of the bonds and molecular fragments involved. The assignments were organized according to the main spectral regions: ν(C=O) of the carboxyl group, ν(C=C) of the aromatic ring and conjugated chain, phenolic ν(C–O), δ(O–H), ring deformation modes, and vibrations associated with the methoxy group in trans-ferulic acid.

The discussion of spectral shifts was conducted with consideration of structural differences among the molecules. In particular, p-coumaric acid was compared with caffeic acid to evaluate the effect of additional hydroxylation; p-coumaric acid was compared with trans-ferulic acid to analyze the effect of methoxylation; and the hydroxycinnamic acids were compared with gallic acid to differentiate the influence of the conjugated side chain from that of a highly hydroxylated hydroxybenzoic system.

### 3.9. Multivariate Analysis of Raman Spectra

Principal component analysis (PCA) was applied separately to the solid-state and solution Raman datasets [49]. Each PCA model contained 20 observations, corresponding to five replicate spectra for each of the four phenolic acids. Raman intensities recorded between 400 and 1800 cm^−1^ were used as variables. Before analysis, all spectra were subjected to the same baseline correction, smoothing, spectral cropping, and vector-normalization procedure described in Section 3.5. PCA was performed using OriginPro 2026 software (OriginLab Corporation, Northampton, MA, USA) based on the covariance matrix. The variables were mean-centered, and no additional autoscaling was applied because the spectra had previously been vector-normalized. PC1–PC2 score plots were used exclusively for exploratory visualization of spectral similarities, differences, and within-group dispersion. No supervised classification, external validation, or assessment of predictive performance was performed.

The ellipses shown in the score plots represent 95% confidence regions calculated from the PC1 and PC2 scores of the replicate spectra for each compound. Their size and orientation reflect the within-group covariance structure in the two-dimensional score space. Because only five replicate spectra were available per compound, the ellipses were interpreted descriptively and not as evidence of statistically validated class separation.

### 3.10. Structure–Spectrum Comparison Criteria

The final interpretation was based on the correlation between molecular structure and Raman response. Hydroxylation was mainly evaluated through changes in bands associated with ν(C–O), δ(O–H), and coupled aromatic ring modes. Methoxylation was analyzed from the contributions of the –OCH_3_ group, especially in the C–O and C–H vibrational regions. π-Conjugation was discussed using bands associated with ν(C=C) of the aromatic ring and the C=C–COOH side chain. Finally, differences in the carbonyl region were used to analyze the influence of the electronic environment of the carboxylic group in each phenolic structure.

## 4. Conclusions

This study provided a comparative Raman, DFT, and exploratory chemometric characterization of p-coumaric, caffeic, trans-ferulic, and gallic acids. The experimental spectra showed that the principal vibrational regions retain identifiable contributions from aromatic, C=C, C=O, C–O, O–H, and methoxy-containing coordinates. The hydroxycinnamic acids shared prominent bands associated with the aromatic–C=C–COOH framework, whereas gallic acid exhibited a distinct hydroxybenzoic fingerprint dominated by ring, phenolic, and carboxylic contributions.

The isolated-molecule DFT calculations supported the qualitative assignment of the principal Raman bands and indicated that many features involve coupled normal modes rather than completely localized bond vibrations. MEP, frontier-orbital, and LOL analyses provided complementary information on electrostatic distribution, electronic delocalization, and localization around oxygenated groups. These descriptors were used as qualitative support and not as direct quantitative predictors of Raman intensity.

Solid-state spectra provided clearer exploratory PCA separation than the solution spectra, whereas the ethanol/water medium produced greater broadening, overlap, and attenuation of several oxygen-sensitive bands. The interpretation is limited by the use of four non-factorially selected compounds, one isolated conformer per molecule, and the absence of explicit crystal-packing and solvent models. Therefore, the findings should be regarded as comparative structure–spectrum trends for the selected phenolic acids rather than as a complete isolation of hydroxylation, methoxylation, or conjugation effects.

## Figures and Tables

**Table 1:** Selected structural parameters of the DFT-optimized phenolic acids.

Molecule	C=O Bond (Å)	Carboxylic C–OH Bond (Å)	Phenolic C–O Bond(s) (Å)	Methoxy Bond(s) (Å)	Side-Chain C=C Bond (Å)	Ring–Side-Chain Dihedral (°)
p-Coumaric acid	1.203	1.349	1.355	—	1.334	−179.99
Caffeic acid	1.203	1.349	1.350–1.368	—	1.334	179.76
trans-Ferulic acid	1.203	1.350	1.349	Ar–OCH_3_: 1.362; O–CH_3_: 1.412	1.335	−179.78
Gallic acid	1.202	1.346	1.353–1.367	—	—	—

**Table 2:** Experimental solid-state and solution Raman bands, solid-to-solution shifts, scaled DFT frequencies, and tentative vibrational assignments of the studied phenolic acids.

Molecule	Solid Raman (cm^−1^)	Solution Raman (cm^−1^)	Δν (cm^−1^)	Scaled DFT (cm^−1^)	Assignment	Interpretation
p-Coumaric acid	1603.9	1604.8	+0.9	1603.2	Aromatic/conjugated ν(C=C)	Nearly invariant band associated with the conjugated hydroxycinnamic backbone, indicating minimal solvent perturbation.
p-Coumaric acid	1634.7	1629.9	−4.8	1629.3	Coupled ν(C=C)/ν(C=O)	Small downshift consistent with solvent interaction involving the conjugated C=C–COOH fragment.
p-Coumaric acid	1168.0	1172.8	+4.8	1158.1	ν(C–O) + δ(O–H)	Slight upshift indicating sensitivity of the phenolic region to hydrogen-bonding interactions.
p-Coumaric acid	1256.7	1271.2	+14.5	1244.9	ν(C–O) + ring/O–H coupled mode	Noticeable upshift associated with a solvent-sensitive phenolic C–O/O–H contribution.
p-Coumaric acid	1446.7	1452.5	+5.8	1439.9	Aromatic ring/C–H deformation	Small displacement; partial overlap with solvent-associated deformation bands cannot be excluded.
Caffeic acid	1611.6	1604.8	−6.8	1618.0	Aromatic/conjugated ν(C=C)	Moderate downshift suggests solvent-mediated perturbation of the hydroxycinnamic framework.
Caffeic acid	1297.2	1277.0	−20.2	1298.7	ν(C–O) + δ(O–H)	Clear downshift consistent with hydrogen bonding involving the catechol-type hydroxyl groups.
Caffeic acid	1181.5	1205.6	+24.1	1183.8	ν(C–O)/δ(O–H) coupled mode	Pronounced upshift reflecting changes in the local hydrogen-bonding environment of phenolic groups.
Caffeic acid	1104.4	1084.1	−20.3	1131.2	Aromatic C–H in-plane/ring mode	Downshift that may reflect solvent perturbation and partial overlap with ethanol C–O vibrations.
Caffeic acid	1451.5	1452.5	+1.0	1447.3	Aromatic ring deformation	Nearly invariant ring-associated band, although a minor solvent contribution may be present.
trans-Ferulic acid	1601.0	1600.0	−1.0	1615.1	Aromatic/conjugated ν(C=C)	Nearly unchanged marker of the conjugated hydroxycinnamic backbone.
trans-Ferulic acid	1628.9	1630.0	+1.1	1625.1	Coupled ν(C=C)/ν(C=O)	Minimal shift, indicating that the conjugated carboxylic mode remains identifiable in solution.
trans-Ferulic acid	1271.2	1270.0	−1.2	1256.4	ν(C–OCH_3_) + ν(C–O)	Nearly unchanged methoxy/phenolic C–O contribution, remaining spectrally detectable in solution.
trans-Ferulic acid	1176.7	1190.0	+13.3	1175.0	ν(C–O) + δ(O–H)	Upshift consistent with solvent interaction involving phenolic and carboxylic groups.
trans-Ferulic acid	1430.3	1450.0	+19.7	1429.7	Aromatic ring/methoxy-coupled deformation	Apparent upshift that may also contain contributions from overlapping ethanol/water bands.
trans-Ferulic acid	813.2	801.0	−12.2	810.1	Ring deformation/C–H out-of-plane mode	A moderate downshift indicates the sensitivity of the low-wavenumber ring mode to the surrounding medium.
Gallic acid	1739.8	1723.4	−16.4	1781.4	ν(C=O) of carboxylic acid	Significant downshift consistent with solvent interaction involving the carboxylic group.
Gallic acid	1300.1	1274.1	−26.0	1299.9	Phenolic ν(C–O) + δ(O–H)	A clear downshift is associated with the hydrogen bonding of the multiple phenolic hydroxyl groups.
Gallic acid	1137.2	1083.2	−54.0	1159.2	Ring + ν(C–O) coupled mode	Large apparent downshift; partial overlap with solvent C–O vibrations is likely.
Gallic acid	1413.9	1451.5	+37.6	1399.2	Aromatic ring deformation	Large apparent upshift that may be influenced by overlap with an ethanol/water band.
Gallic acid	843.1	875.9	+32.8	858.6	Ring deformation/out-of-plane mode	Apparent displacement indicates environmental sensitivity, although solvent overlap cannot be excluded.

Note: Δν = νsolution − νsolid. Positive values indicate an upshift in solution, whereas negative values indicate a downshift. Assignments are tentative and were established from the combined analysis of experimental band positions, scaled DFT frequencies, and calculated normal-mode character. Solution bands may contain solvent-induced shifts and partial overlap with contributions from the ethanol/water mixture.

**Table 3:** Frontier orbital energies and electronic descriptors of the studied phenolic acids.

Molecule	EHOMO (eV)	ELUMO (eV)	ΔEgap (eV)	Dipole Moment (D)	Isotropic Polarizability, αiso (a.u.)
p-Coumaric acid	−7.58	−1.00	6.58	2.64	124.16
Caffeic acid	−7.40	−1.04	6.36	1.80	129.23
trans-Ferulic acid	−7.33	−1.00	6.33	3.93	142.13
Gallic acid	−7.75	−0.38	7.37	2.22	98.00

Note: ΔEgap was calculated as ELUMO − EHOMO. The isotropic polarizability was obtained from the diagonal components of the calculated polarizability tensor.

**Table 4:** Instrumental conditions used for the acquisition of the experimental Raman spectra.

Instrumental Parameter	Experimental Condition
Spectrometer model	DXR3 Raman Microscope
Manufacturer	Thermo Scientific, Thermo Fisher Scientific (Waltham, MA, USA)
Excitation laser wavelength	532 nm
Laser power	7.0, according to the instrumental power setting
Microscope objective	MPlan 10×/0.25 BD
Confocal aperture	50 µm
Estimated laser spot diameter	2.1 µm
Diffraction grating	900 grooves mm^−1^
Spectral resolution	1.7–2.7 cm^−1^
Spectral step used in the processed dataset	1.0 cm^−1^
Instrumental acquisition range	50–3500 cm^−1^
Spectral range used for vibrational analysis	400–1800 cm^−1^
Integration time	1.0 s per exposure
Number of exposures/accumulations	64
Total acquisition time	64 s per spectrum
Number of background exposures	64
Number of replicate spectra	Five spectra per compound and physical state
Calibration standard	Silicon band at 520.7 cm^−1^
Solid-sample holder	Glass microscope slide
Solution-sample holder	Glass Raman vial
Acquisition software	OMNICxi 3.0 software, Thermo Scientific
Statistical processing software	OriginPro 2026, OriginLab Corporation

## Data Availability

The original contributions presented in this study are included in the article. Further inquiries can be directed to the corresponding authors.
